# Effects of vitamin D supplementation on serum lipid profile in women with polycystic ovary syndrome: A protocol for a systematic review and meta-analysis

**DOI:** 10.1097/MD.0000000000020621

**Published:** 2020-06-05

**Authors:** Xiao-yan Shi, Jia Yao, Si-min Fan, Pei-pei Hong, Yu-guo Xia, Qiu Chen

**Affiliations:** Hospital of Chengdu University of Traditional Chinese Medicine, Chengdu, Sichuan Province, P.R. China.

**Keywords:** polycystic ovary syndrome, protocol, serum lipid profile, systematic review, vitamin D supplementation

## Abstract

**Background:**

Polycystic ovary syndrome (PCOS) is the commonest endocrine disorder in reproductive-aged women. In addition to the reproductive consequences, PCOS is also characterized by a metabolic disorder, which may play a part in the etiology of anovulation and has important implications for long-term health as well. Vitamin D deficiency is prevalent in PCOS and there is a close relationship between metabolic dysfunction and vitamin D status in women with PCOS. The purpose of this systematic analysis is to evaluate the effect of vitamin D supplementation on serum lipid profiles in patients with PCOS.

**Methods:**

We will search five databases for relative studies: Medline, the Cochrane Library, EMBASE, Web of Science, and ClinicalTrials.gov and identified all reports of randomized controlled trials published prior to July 2020. Two authors will independently scan the articles searched, extract the data from articles included, and assess the risk of bias by Cochrane tool of risk of bias. Disagreements will be resolved by discussion among authors. All analysis will be performed based on the Cochrane Handbook for Systematic Reviews of Interventions. Fixed-effects model or random-effects model was used to calculate pooled estimates of weighted mean difference (WMD) with 95% confidence intervals.

**Results:**

This review will be to assess the effect of vitamin D supplementation on serum lipid profiles in patients with PCOS. The results of the study will be published in a scientific journal after peer-review.

**Conclusions:**

These findings will provide guidance to clinicians and patients on the use of vitamin D for PCOS with dyslipidemia.

**Ethics and dissemination:**

This study is a protocol for a systematic review of vitamin D as a treatment of dyslipidemia in PCOS patients.

**Systematic review registration:**

INPLASY202050007.

## 1 Introduction

Polycystic ovary syndrome (PCOS) is the commonest endocrine disorder affecting 5% to 20% of reproductive-aged women, and the majority cases of anovulatory infertility and of hirsutism.^[1,2]^ In addition to the reproductive consequences, PCOS is also characterized by a metabolic disorder, which may play a part in the etiology of anovulation and has important implications for long-term health as well.^[3–6]^ Among PCOS-related metabolism dysfunction, dyslipidemia is certainly highly prevalent. According to the National Cholesterol Education Program (NCEP) guidelines,^[7]^ as many as 70% of women with PCOS exhibit abnormal serum lipid concentrations. Even in lean women of PCOS, a higher prevalence of atherogenic lipid profile was demonstrated.^[8]^ Women with PCOS have androgen excess, insulin resistance, variable amounts of estrogen exposure, and many environmental factors, all of which can influence lipid metabolism.^[9,10]^ Furthermore, as the common frequent but modifiable metabolic disturbances, dyslipidemia could exaggerate the risk for atherosclerosis and cardiovascular disease of PCOS patients.^[11]^ Thus, the treatment of dyslipidemia should be incorporated into the routine PCOS subjects’ wellness care program.^[12]^ So far, statins and fibrates are the most common hypolipidemic drugs, however, their efficacy to achieve normal concentrations of lipids is limited, besides it has been observed that both statins and fibrates have adverse effects including hepatotoxicity and myopathy.^[13,14]^ Taking this into account, identifying new strategies like complementary agents with lipid-improving properties, which can be used alongside low doses of statins, has attracted a lot of interest.^[15–17]^

Vitamin D deficiency is a worldwide problem that may affect up to half of the general adult population, and it is even more prevalent in PCOS patients. Numberous studies have suggested that there is an association between vitamin D status and metabolic dysfunctions (insulin resistance, androgen excess and dyslipidemia) in women with PCOS. Vitamin D for years was known as a key hormone involved in the regulation of bone growth and calcium/phosphorous balance.^[18]^ Beyond the skeletal effects, the role of vitamin D in the regulation of lipid metabolism has recently come into notice. It has been suggested that vitamin D may decrease hepatic triglycerides (TG) production or secretion via its effects on calcium intake and increase the clearance of circulating lipoprotein particles by activating the lipoprotein lipase (LPL).^[19–20]^ Furthermore, vitamin D status could alter the balance between pro- and anti-inflammatory cytokines and thus affect lipid metabolism (correlated with improvement in insulin resistance).^[21]^ Observational studies also reported an inverse correlation between higher concentrations of serum 25-hydroxy cholecalicferol (25(OH)D) and lower concentrations of total cholesterol (TC), TG, low-density lipoprotein cholesterol (LDL-C), and higher concentrations of high-density lipoprotein cholesterol.^[22–24]^ And various clinical trials have assessed the effects of vitamin D supplementation on circulating lipids concentrations among PCOS patients,^[25–27]^ however, the results are conflicting: with some studies demonstrating the positive effects on circulating lipid concentrations, while others showing no beneficial effects. Given that available published randomized clinical trials (RCTs) have an amount of uncertainty regarding the effect of vitamin D supplementation on serum lipid profile and considering the point that these studies are limited in sample size, a systematic review and meta-analysis would be appropriate to resolve the current controversy and reach a conclusive result for the effect of vitamin D supplementation on serum lipid profile.

The objective of the current systematic review and meta-analysis is to explore the effect of vitamin D supplementation on serum lipid profile in women with PCOS, based on data available in RCTs. And these findings may provide guidance to clinicians and patients on the use of vitamin D for PCOS with dyslipidemia.

## 2 Methods

### 2.1 Registration

Our meta-analysis protocol has been registered in the International platform of registered systematic review and meta-analysis protocols (INPLASY) as number INPLASY202050007.

This study are designed in accordance with the Preferred Reporting Items for Systematic Reviews and Meta-analysis guidelines.^[28]^

### 2.2 Eligibility criteria

We will include studies according to the following inclusion criteria:

(1) study design: RCTs with any follow-up duration and sample size were allowed;
(2) population: patients of any age or ethnic origin with a definitive diagnosis of PCOS;
(3) intervention: vitamin D at any dose and route;
(4) control: placebo;
(5) outcomes: TC, TG, LDL-C, very LDL-C, high-density lipoprotein cholesterol.

### 2.3 Search methods for the identification of studies

Two authors (XS and JY) will independently search databases including Medline, the Cochrane Library, EMBASE, and Web of Science until July 2020. According to the PICOS principle, the keywords of our search terms were: (“vitamin D” OR “cholecalciferol” OR “25-hydroxyvitamin D2” OR “24, 25-dihydroxy vitamin D3”) AND (“polycystic ovary syndrome” OR “PCOS”). The ClinicalTrials.gov registry will also be searched for unpublished trials and the authors will be contacted for any additional information if necessary. Relevant references from included studies will be sought to retrieve additional eligible studies.

### 2.4 Data collection

#### 2.4.1 Study selection

**Figure 1:**
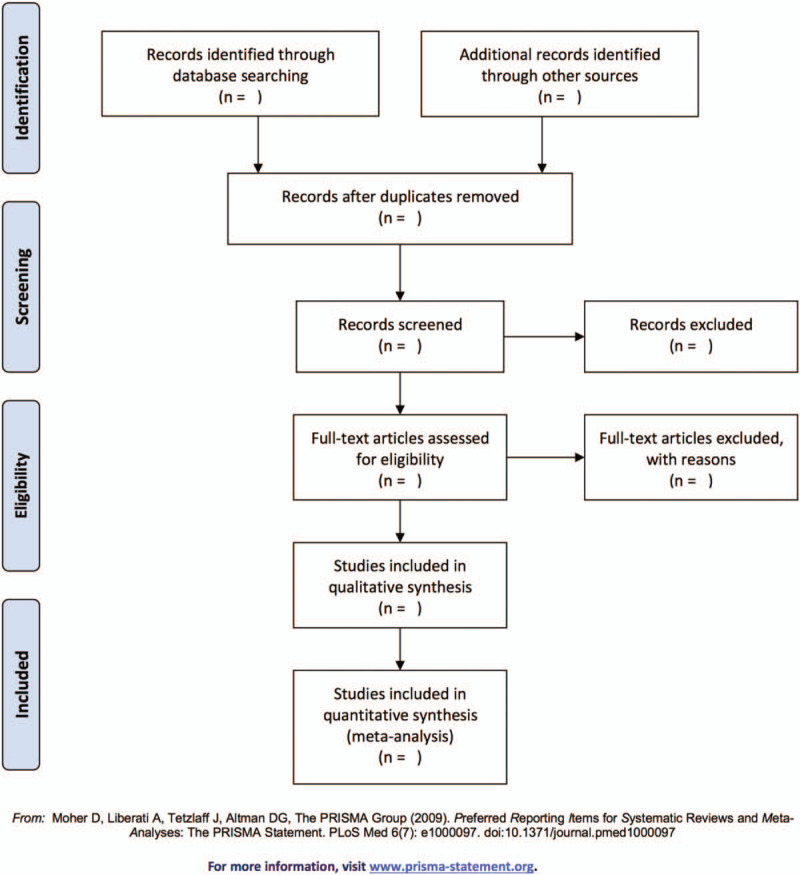
Flow diagram of study selection.

Basing on the eligibility criteria, two reviewers (XS and JY) will independently review all identified data. Duplicate literature will be removed. The full text of the articles will be retrieved for review if 1 or more reviewers deemed the studies be included or when they are uncertain about the inclusion after they read the abstracts. Researches that both reviewers judge to be irrelevant will be filtered out. A third reviewer (SF) will consult for resolutions of any disagreements. The selection process will be shown in a Preferred Reporting Items for Systematic Review and Meta-analysis (PRISMA) flow chart (Fig. 1).

#### 2.4.2 Data extraction

Two reviewers (XS and JY) will perform the data extraction, and a third viewer (SF) will be involved in a discussion for any disagreements. The following information of eligible articles will be extracted to a data extraction form: author, year of publication, sample size, mean age, doses of vitamin D, follow-up duration, study design, body mass index (BMI), mean serum 25OHD (ng/mL) of the subjects and outcomes.

### 2.5 Quality assessment

Based on the Cochrane Handbook for Systematic Reviews (version 5.3.0),^[29]^ we will assess the methodological quality of all studies. The risks of bias will be classified as low, unclear, or high by evaluating the 7 components as random sequence generation, allocation concealment, blinding of outcome assessment, blinding of participants and personnel, incomplete outcome data, selective outcome reporting, and other bias. Two independent reviewers (XS and JY) will conduct this assessment, and a third reviewer (SF) will be consulted for any disagreements.

### 2.6 Data analysis

#### 2.6.1 Measurement of the treatment effect

We will calculate the WMD and 95% confidence intervals of all outcomes (TC, TG, LDL, high-density lipoprotein, very LDL-C).

#### 2.6.2 Dealing with missing data

If raw data are not directly provided in the text, tables, or figures in the study will be referred to. Once relevant details are insufficiently reported in studies, authors will be contacted and the ClinicalTrials.gov register will be searched for further information.

#### 2.6.3 Assessment of heterogeneity

Study heterogeneity will be tested by *χ*2-based Cochran *Q* statistic and *I*^2^ statistic (*P* value < .10 or *I*^2^ statistic >50% indicated significant heterogeneity). The random-effects model (inverse variance method) of analysis will be used to pool the estimations of WMD across studies if significant heterogeneity is detected. In other cases, the fixed-effects model (inverse variance method) will be employed.

#### 2.6.4 Assessment of reporting biases

Using the funnel plot, Egger and Begg test to judge publication bias. In terms of accuracy, the Funnel plot is not as good as Egger test and Begg test, while Begg test is not as sensitive as Egger test. When the 3 results are inconsistent, first give up the Funnel plot. When the Egger test and the Begg test result are opposite, the result of the Egger test will be used as the result. And the trim-and-fill method will be performed to adjust for publication bias in meta-analysis.^[30]^

#### 2.6.5 Subgroups analysis and sensitivity analysis

Subgroup analysis will be performed based on vitamin D doses, intervention duration, and type of supplementation. We will remove the included studies 1 by 1 to evaluate the reliability of the results of the meta-analysis for a sensitivity analysis.

## 3 Discussion

PCOS is the commonest endocrine disorder in women. The metabolic disorder, especially dyslipidemia and vitamin D deficiency are prevalent in PCOS women and there is a close relationship between metabolic dysfunction and vitamin D status in women with PCOS. Taking this into account, there are rational premises for supplementing PCOS patients with vitamin D. Moreover, a recent meta-analysis of Lagowska K et al^[31]^ has suggested that there are positive effects of vitamin D supplementation on insulin resistance in women with PCOS. In our present study, we will comprehensively and systematically review the currently available evidence to investigate the effects of vitamin D supplementation on blood lipid parameters in PCOS patients. These findings will provide guidance to clinicians and patients on the use of vitamin D for PCOS with dyslipidemia.

## Author contributions

**Conceptualization:** Xiao-yan Shi, Qiu Chen.

**Data analysis:** Si-min Fan, Xiao-yan Shi.

**Data extraction:** Jia Yao, Xiao-yan Shi.

**Funding acquisition:** Yu-guo Xia.

**Methodology:** Qiu Chen.

**Project administration:** Qiu Chen.

**Resources:** Qiu Chen.

**Software:** Xiao-yan Shi, Si-min Fan.

**Writing – original draft:** Xiao-yan Shi, Jia Yao, Yu-guo Xia.

**Writing – review & editing:** Xiao-yan Shi, Pei-pei Hong.
